# Escin Attenuates Amyloid Beta 1‐42‐Induced Oxidative Stress, Apoptosis, and Neuroinflammation in Neuron‐Like SH‐SY5Y Cells

**DOI:** 10.1002/jbt.70903

**Published:** 2026-05-13

**Authors:** Sakine Akar, Ozge Alvur, Gulsah Evyapan, Berna Ozdem, Ilknur Porsuk Doru

**Affiliations:** ^1^ Department of Medical Biology, Faculty of Medicine Van Yuzuncu Yıl University Van Turkey; ^2^ Department of Medical Biology and Genetics, Health Science Institute Inonu University Malatya Turkey

**Keywords:** Alzheimer's disease, apoptosis, Aβ1‐42, Escin, NF‐κB signaling, oxidative stress, SH‐SY5Y cells

## Abstract

The pathogenesis of Alzheimer's disease (AD) involves amyloid beta (Aβ)‐induced oxidative stress, apoptotic cell death, and neuroinflammation, contributing to neuronal dysfunction. In our study, a differentiation protocol using retinoic acid was applied to SH‐SY5Y cells to generate a neuron‐like phenotype, and the neuroprotective efficacy of Escin was investigated by inducing Aβ1‐42‐mediated cytotoxicity. The experimental protocol involved an initial treatment with 2 µM Escin prior to Aβ1‐42 application. Cell viability, intracellular reactive oxygen species (ROS), apoptosis, and inflammatory mediator expression (NF‐κB, TNF‐α, IL‐1β) were assessed by MTT assay, flow cytometry with DCFH‐DA, flow cytometry with Annexin V‐FITC/7‐AAD staining, and RT‐qPCR, respectively. In our results, Aβ1‐42 exposure was found to significantly reduce cell viability and increase ROS production. Additionally, it was observed to enhance apoptotic cell death and increase pro‐inflammatory gene expression. Escin pretreatment was found to significantly mitigate these effects by reducing oxidative stress, apoptosis, and NF‐κB‐mediated inflammatory signaling. Furthermore, galantamine (10 µM), an approved AD treatment agent, was used as a positive control to compare the effects of Escin and confirmed the experimental model by exhibiting protective effects. In conclusion, these findings demonstrate that Escin is a promising neuroprotective agent and warrant further investigation into its potential to mitigate Aβ‐related neuronal damage in AD.

## Graphical abstract

This study demonstrates that Escin pretreatment exerts potent neuroprotection against Aβ1‐42‐induced toxicity in differentiated SH‐SY5Y cells. By suppressing the NF‐κB inflammatory axis, mitigating oxidative stress, and inhibiting the apoptotic cascade, Escin provides a mechanistic basis for its potential as a therapeutic candidate for Alzheimer's disease.

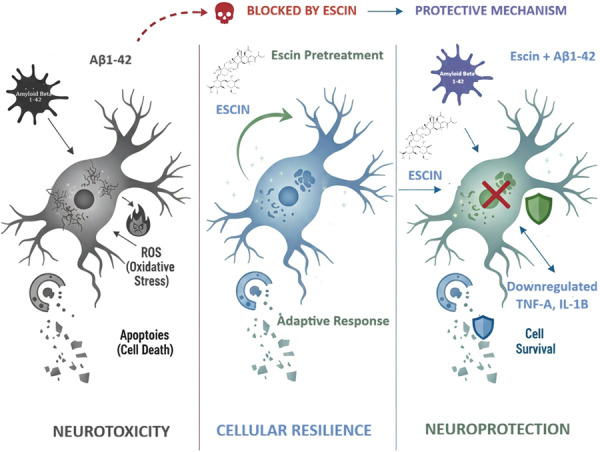

## 1 Introduction

Alzheimer's disease (AD), which develops in advanced ages, is a neurological disorder that causes losses in memory and cognitive functions, adversely affecting an individual's daily life [1, 2]. The pathophysiology of this disease involves both senile plaques caused by misfolded amyloid‐beta (Aβ) peptides accumulating extracellularly and neurofibrillary tangles formed by hyperphosphorylated tau proteins accumulating intracellularly [2]. These alterations primarily impact brain regions like the hippocampus and cortex, leading to synaptic failure, neuroinflammation, and neuronal loss [3].

Aβ plays multiple roles in AD pathology, including neurotoxicity [4], dysregulation of intracellular calcium balance [5], and the induction of synaptic dysfunction [6]. Aβ peptides differ in their neurotoxic effects depending on their length and aggregation properties. Compared to other isoforms, Aβ1‐42 demonstrates increased amyloidogenicity and cytotoxicity, owing to its enhanced aggregation potential and tendency to form fibrils [7]. Multiple in vitro and in vivo investigations have validated that Aβ1‐42 contributes to neuronal degeneration by triggering pathways such as oxidative stress, mitochondrial impairment, neuroinflammation, and apoptosis [8, 9].

Escin is a triterpenoid saponin extracted from the seeds of *Aesculus hippocastanum* (horse chestnut) [10]. Traditionally, Escin has been used to treat edema, vascular insufficiency, and inflammatory conditions [11]. Recent studies have validated these uses and highlighted its broad pharmacological effects. The selection of Escin for this neuroprotective study is driven by its multi‐target pharmacological profile and its potential for clinical translatability. Beyond its lipophilic triterpenoid structure, which suggests a capacity for functional interaction with the central nervous system (CNS), Escin exhibits unique corticosteroid‐like anti‐inflammatory activity by modulating the glucocorticoid receptor (GR)/p38 MAPK/NF‐κB signaling axis, providing potent protection without the typical systemic side effects of synthetic steroids [12]. Recent evidence further underscores its clinical relevance in neurodegenerative contexts; Escin not only stabilizes the blood‐brain barrier (BBB) integrity by upregulating tight junction proteins such as ZO‐1 and Occludin via the AMPK/Cav‐1/MMP‐9 pathway [13, 14], but also exerts indirect neuroprotection through the gut‐brain axis. Specifically, Escin has been shown to attenuate secondary brain injury by maintaining intestinal barrier function and preventing the systemic leakage of lipopolysaccharides (LPS), thereby mitigating neuroinflammation even in scenarios of limited direct brain penetration [15, 16]. A wide range of studies has highlighted Escin's strong antioxidant and anti‐inflammatory capabilities, which are primarily linked to its modulation of pathways involved in oxidative stress and immune regulation [17, 18]. The anti‐inflammatory mechanism of Escin is primarily characterized by its ability to modulate the NF‐κB transcription complex, which in turn suppresses the downstream liberation of various mediators of inflammation, most notably tumor necrosis factor‐alpha (TNF‐α) and interleukin‐1 beta (IL‐1β) [12, 19]. It also helps maintain mitochondrial integrity, thereby preventing apoptosis triggered by mitochondrial damage in various diseases [18, 20].

Increasing evidence indicates that Escin exerts neuroprotective actions, particularly by limiting neuroinflammation and oxidative stress in neuronal injury models, including those associated with Parkinsonian neurotoxicity [21, 22]. Importantly, since Aβ‐induced neuronal injury is strongly driven by oxidative stress, mitochondrial dysfunction, and inflammatory responses, Escin represents a rational pharmacological candidate due to its capacity to modulate these critical AD‐related pathogenic mechanisms through its multi‐target lipophilic profile. However, despite its well‐documented biological potential, there is currently no study directly evaluating the neuroprotective effects of Escin in Aβ‐induced cellular models of AD. This gap underscores the need to determine whether Escin can counteract Aβ‐associated oxidative, apoptotic, and inflammatory processes contributing to AD‐related neuronal injury. Moreover, existing evidence suggests that Escin may support neuronal survival through multiple protective mechanisms [17, 18, 19, 23].

Galantamine, a natural alkaloid from the Amaryllidaceae family, is FDA‐approved for Alzheimer's disease treatment as an acetylcholinesterase inhibitor, enhancing cholinergic neurotransmission. It also exerts neuroprotective effects by scavenging ROS, reducing oxidative stress–induced DNA damage, and modulating inflammatory responses, while promoting non‐amyloidogenic processing of amyloid precursor protein [24, 25].

This research is designed to investigate the hypothesis that Escin can alleviate Aβ1‐42‐induced oxidative damage, programmed cell death, and neuroinflammation in SH‐SY5Y cells with neuron‐like phenotype acquired by RA. Additionally, Galantamine is included as a reference point to provide a comparative perspective on established neuroprotective mechanisms related to AD. This study, focusing on fundamental cellular processes in an in vitro SH‐SY5Y cell model of AD, allows us to understand the potential of Escin as a therapeutic candidate in AD‐associated neurodegeneration.

## 2 Materials and Methods

### 2.1 Drug Treatment

Differentiated SH‐SY5Y cells were subjected to pharmacological treatments following completion of the retinoic acid induced differentiation protocol. A stock solution of Escin (≥ 98% purity; USP, Catalog No: 1249202, Singapore) was formulated by dissolving the compound in dimethyl sulfoxide (DMSO; Sigma‐Aldrich, USA). This stock was then appropriately diluted with the culture medium to reach the desired working doses, maintaining the final DMSO concentration at a safe threshold of less than 0.1% (v/v) to avoid vehicle‐induced toxicity. Cells were pretreated with Escin (2 µM) for 24 h (h) prior to amyloid‐beta exposure.

Following established aggregation protocols, Aβ1‐42 peptide (A9810; Sigma‐Aldrich, USA) was prepared in HFIP (Sigma‐Aldrich, USA) and incubated accordingly. The resulting aggregated peptides were then administered to the cells at a dose of 10 µM; this 24 h treatment was utilized to trigger the desired cytotoxic response. Galantamine (Cayman Chemical, Cat. No: 17559, USA) was employed as a positive control at 10 µM and administered 24 h before Aβ1‐42 treatment, based on previous studies, to provide a reference for established AD‐relevant neuroprotective effects and to enable direct comparison with Escin [26, 27].

Experimental groups included untreated differentiated, Escin alone, Aβ1‐42 alone, Escin (Esc)+ Aβ1‐42, and Galantamine (Gal) + Aβ1‐42.

### 2.2 Cell Growth Conditions

To simulate AD like conditions in vitro, we employed SH‐SY5Y neuroblastoma cells (Sigma‐Aldrich, USA), selecting this line for its potential to develop neuron‐like features. The growth environment consisted of MEM (MEM; Simply Biologics, Cat. No: CC136‐1000, USA) supplemented with 10% FBS (Capricorn Scientific, Cat. No: FBS‐10A; Germany), 2 mM l‐glutamine, and 1% penicillin–streptomycin (Capricorn Scientific, Cat. No: P0781; Germany). Incubation took place in T‐25 flasks under standard conditions of 37°C and 5% CO₂. For the sake of experimental consistency and cell health, the line was passaged regularly upon achieving 80% density.

### 2.3 Cell Differentiation

To promote a cholinergic neuron‐like phenotype, SH‐SY5Y neuroblastoma cells underwent a differentiation process. This was achieved by adjusting the growth medium to include 10 µM all‐trans retinoic acid and simultaneously decreasing FBS content to 1% [28]. Cells were maintained under these conditions for 14 days. During the differentiation period, the medium was refreshed every 3 days, consistently containing 1% FBS and 10 µM retinoic acid (RA) (Cat. No: R2625; Sigma‐Aldrich, USA). To verify successful differentiation, morphological evaluation was conducted, and neurite extension was quantitatively assessed by microscopic examination.

### 2.4 Morphological Analyses

Morphological alterations among the experimental groups were evaluated using an inverted microscope (Zeiss Axio, Germany) equipped with a 40× objective lens. Neurite outgrowth parameters were assessed using ImageJ software, where both neurite length and neurite count were manually quantified. For each condition, ten cells from randomly selected microscopic fields were analyzed. The obtained measurements were expressed as the percentage of total neurite length or neurite number within each treatment group.

### 2.5 Evaluation of Cell Viability

To initiate the assays, 96 well plates were used to seed the differentiated neuron‐like cells at a concentration of 5 × 10³ cells/well, followed by a 24 h attachment period. To identify the optimal dose and potential toxicity, we exposed the cells to varying ranges of Aβ1‐42 (0.5–20 µM) and Escin (0.5–10 µM) for 24 h. Based on these preliminary dose‐response analyses, 10 µM for Aβ1‐42 (representing the IC₅₀) and a non‐toxic concentration of 2 µM for Escin were selected for all following procedures

In the experimental phase, cells were initially preincubated with Escin for 24 h, followed by a subsequent 24 h treatment with Aβ1‐42. The survival of the cells was then determined through an MTT assay (Cayman Chemical, Cat. No: 10009591), following the provided laboratory guidelines. To each well, 20 µL of the MTT reagent (prepared at 5 mg/mL) was introduced and kept at 37°C for 4 h. After the resulting formazan crystals were fully dissolved, the optical density was recorded at 540 nm with an Azure Biosystems microplate reader (AC3000, USA). Results were normalized and presented as a percentage of the untreated control cells.

### 2.6 Determination of Oxidative Stress

The levels of internal ROS were evaluated through flow cytometry using the H2DCFDA probe (ABP Bioscience, Cat. No: A057). Once the drug treatments were completed on the seeded cells (3 × 10⁵ per sample), the cultures were collected and resuspended in PBS containing 10 µM H2DCFDA. A 20 min (min) incubation at 37°C allowed for proper dye uptake. To validate the assay, 200 µM TBHP was utilized for 30 min as a positive control. Fluorescence intensity, which corresponds to the ROS concentration, was measured with a 488 nm excitation using the Beckman Coulter Cytoflex system. The baseline for all final calculations was established using the untreated control group, with results presented as a relative percentage.

### 2.7 Detection of Apoptosis

The Annexin V‐FITC/7‐AAD dual staining method (Cat. No. 640922, BioLegend, USA) was employed to evaluate the extent of apoptosis in differentiated SH‐SY5Y cultures. Once cells (3 × 10⁵/well) had undergone the specified pharmacological treatments, they were harvested and processed according to the kit's instructions. A 15 min incubation in a dark, room‐temperature environment followed the addition of the staining reagents. SH‐SY5Y cells that had been differentiated to exhibit neuronal characteristic was immediately performed on a Beckman Coulter Cytoflex Flow Cytometer. The results were expressed as a ratio compared to the baseline values of the differentiated control samples.

### 2.8 Quantitative Real‐Time PCR (qRT‐PCR)

Following RNA extraction with TRIzol™ (Cat. No. 15596026, Invitrogen, USA) from treated SH‐SY5Y cells, RNA quality was confirmed using A260/A280 ratios (1.8–2.0) on a NanoDrop system. cDNA was then generated from a 1 µg RNA template using the iScript™ kit (Cat. No. 1708891, BioRad, USA) according to a previously described protocol [29]. Quantitative PCR amplification was performed on an Applied Biosystems StepOnePlus (Thermo Fisher Scientific, USA) system using SYBR Green Master Mix (A.B.T. Biosciences, USA) and 100 ng of cDNA per well. The housekeeping gene β‐actin served as an internal control for the relative quantification of TNF‐α, IL‐1β, and NF‐κB mRNA levels. The 2^–ΔΔCt^ equation was applied for calculation, ensuring that all analyzed samples had Ct values below 35. Primer sequences can be found in Table 1.

**Table 1:** Human primers used for RT‐qPCR.

Gene	Primer sequence (5′→3′)
NF‐κB Forward	GAAGCACGAATGACAGAGGC
NF‐κB Reverse	GCTTGGCGGATTAGCTCTTTT
TNF‐α Forward	CCTCTCTCTAATCAGCCCTCTG
TNF‐α Reverse	GAGGACCTGGGAGTAGATGAG
IL1‐β Forward	ATGATGGCTTATTACAGTGGCAA
IL1‐β Reverse	GTCGGAGATTCGTAGCTGGA
β‐actin Forward	CAACCGCGAGAAGATGAC
β‐actin Reverse	AGGAAGGCTGGAAGAGTG

### 2.9 Statistical Analysis

Statistical evaluations were carried out using GraphPad Prism 8.0, with results reported as mean ± SD (*n* = 3). A significance level of *p* < 0.05 was applied across all tests. After verifying data normality (Shapiro–Wilk) and variance homogeneity (Levene's), group differences were analyzed. One‐way ANOVA with Tukey's post hoc correction was used for multiple group comparisons, while Student's t‐test was reserved for comparing two distinct groups and establishing IC₅₀ values.

## 3 Results

### 3.1 Retinoic Acid Promotes Neuronal‐Like Morphological Differentiation in SH‐SY5Y Cells

**Figure 1:**
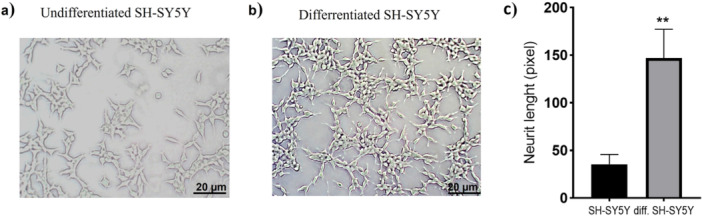
Morphological maturation of SH‐SY5Y cells induced by retinoic acid. a) Control (undifferentiated) cells show compact clusters with negligible neurite extensions. b) RA‐treated cells display neuronal‐like architecture with ramified neurite projections. c) Quantitative metrics confirm a significant expansion in neurite length after differentiation compared to the control population (22 ± 1.2 pixels, *n* = 7 vs 128 ± 20.3 pixels, *n* = 7; *p* < 0.01). Scale bar = 20 µm.

To investigate the effect of escin on Alzheimer's disease, a neuron‐like *in vitro* model, already widely used in the literature, was created using the RA‐induced SH‐SY5Y differentiation protocol. In this model, SH‐SY5Y cells cultured in RA‐containing medium for 14 days showed significant morphological remodeling (Figure 1b). While undifferentiated control cells had a compact and clustered appearance showing minimal neurite development, RA treatment resulted in the formation of long and branched neurite extensions. Quantitative assessments confirmed this phenotypic change and showed a significant increase in neurite length in undifferentiated SH‐SY5Y cells compared to baseline (*p* < 0.01). These results demonstrated that SH‐SY5Y cells successfully transformed into a neuron‐like morphology after RA induction.

### 3.2 Escin Mitigates Aβ1‐42 Induced Cytotoxicity in Neuron‐Like Differentiated SH‐SY5Y Cells

SH‐SY5Y cells, which were given a neuron‐like morphology with RA, were treated with different concentrations of Aβ1‐42 (1–20 μM) and Escin (0.5–10 μM) for 24 h, and cell viability was evaluated using the MTT test, respectively (Figure 1ab). SH‐SY5Y cells treated with Aβ1‐42 showed a significant dose‐dependent decrease in cell viability compared to untreated control cells. Among the evaluated doses, 10 μM Aβ1‐42 caused a pronounced decline in viability relative to the control (***p < 0.001) and was therefore selected for subsequent experiments. Treatment with Escin (0.5–10 μM) showed a dose‐dependent decrease in the percentage of cell viability of SH‐SY5Y cells. Escin was not cytotoxic at concentrations of 0.5, 1, and 2 μM, but showed significantly cytotoxic effects at concentrations of 4 μM (**p < 0.01) and 10 μM (***p < 0.001). Thus, 2 μM Escin was designated as the highest noncytotoxic concentration.

**Figure 2:**
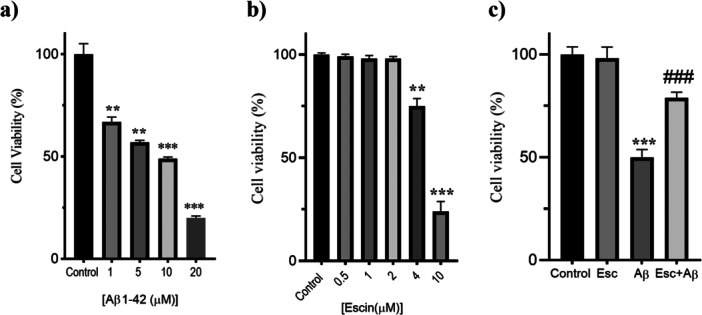
Escin mitigates Aβ1‐42 mediated cytotoxic damage in differentiated SH‐SY5Y cultures. a) Dose‐response analysis of Aβ1‐42 (1–20 μM) on cellular survival over 24 h. b) Impact of various Escin concentrations (0.5–10 μM) on viability to establish safety margins. c) Neuroprotective efficacy of a 2 μM Escin pretreatment against 10 μM Aβ1‐42 induced injury. Survival was quantified via MTT assay and normalized to the baseline of untreated, differentiated controls. Data are presented as mean ± SD (*n* = 3). **p < 0.01 versus control group, ***p < 0.001 versus control group, ^###^ *p* < 0.001 vs. Aβ1‐42 group.

To evaluate the neuroprotective capacity of Escin, differentiated neuron‐like cells were pre‐treated with 2 μM Escin prior to Aβ1‐42 application. This treatment significantly attenuated the Aβ1‐42‐induced decrease in cell viability (Figure 2c). Specifically, the Escin pre‐treated cells exhibited a significantly higher survival rate compared to the group exposed only to Aβ1‐42 (***p < 0.001), demonstrating that Escin provides considerable cytoprotection against Aβ1‐42‐mediated neurotoxicity.

### 3.3 Escin Attenuates the Accumulation of Aβ1‐42 Triggered Reactive Oxygen Species

**Figure 3:**
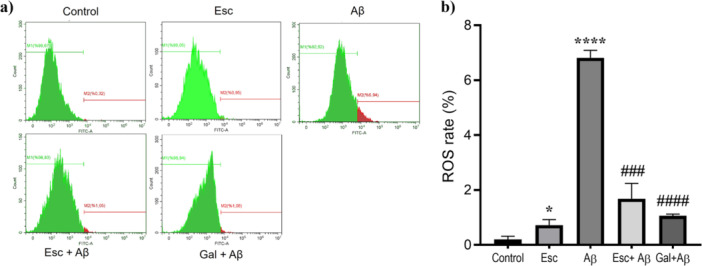
Escin attenuates Aβ1‐42 induced reactive oxygen species (ROS) generation. a) Representative flow cytometry histograms of intracellular ROS levels determined using H2DCFDA staining in neuron‐like differentiated SH‐SY5Y cells. b) Quantitative analysis of ROS production. Aβ1‐42 exposure markedly increased ROS levels compared with the Control group. Escin pretreatment significantly reduced Aβ1‐42 induced ROS elevation, exhibiting a protective profile comparable to the reference drug, galantamine. Data are expressed as mean ± SD (*n* = 3). *p < 0.05 and ****p < 0.0001 versus Control group; ###*p* < 0.001 and ####*p* < 0.0001 versus Aβ1‐42 treated group.

The capacity of Escin to mitigate Aβ1‐42‐mediated oxidative stress was evaluated by monitoring internal ROS fluctuations in neuron‐like differentiated SH‐SY5Y cells, utilizing DCFDA based fluorescence detection on a flow cytometer. As shown in Figure 3, exposure to Aβ1‐42 resulted in a marked elevation of intracellular ROS levels compared with differentiated control cells (****p < 0.0001).

Treatment with Escin alone resulted in a modest but statistically significant alteration in ROS levels relative to neuron‐like differentiated control cells (*p < 0.05). Notably, pretreatment with Escin significantly attenuated Aβ1‐42‐induced ROS accumulation compared with cells exposed to Aβ1‐42 alone (^###^ *p* < 0.001).

Similarly, pretreatment with galantamine, which was included as a positive control, significantly reduced ROS levels in Aβ1‐42 treated cells when compared with the Aβ1‐42 group (^####^ *p* < 0.0001). The extent of ROS suppression observed in the Escin pretreated group was comparable to that achieved with galantamine under the same experimental conditions.

### 3.4 Escin Pretreatment Reduces Aβ1‐42‐Induced Apoptosis in Neuron‐Like Differentiated SH‐SY5Y Cells

**Figure 4:**
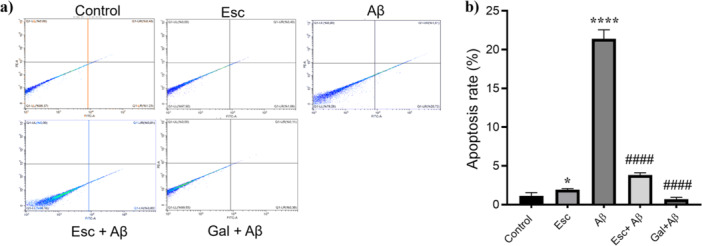
Escin pretreatment reduces Aβ1‐42 induced apoptotic cell death. a) Representative flow cytometry dot plots illustrating apoptotic cell populations (Annexin V‐FITC positive) following Annexin V‐FITC/7‐AAD staining. b) Quantitative analysis of the total apoptotic cell percentages in each group. Data are expressed as mean ± SD (*n* = 3). *p < 0.05 and ****p < 0.0001 versus Control group; ####*p* < 0.0001 versus Aβ1‐42 treated group.

To determine the impact of Escin on Aβ1‐42 triggered programmed cell death, cultures underwent Annexin V‐FITC/7‐AAD dual staining followed by flow cytometric evaluation. Representative dot plots and their corresponding quantitative data are detailed in Figure 4a and b, respectively.

Exposure to Aβ1‐42 triggered a profound rise in the ratio of apoptotic cells relative to the differentiated control group (****p < 0.0001). Escin alone induced a slight yet significant elevation in apoptosis relative to the control (*p < 0.05). Notably, administering Escin before the Aβ1‐42 challenge exerted a substantial inhibitory effect on programmed cell death, leading to a dramatic decline in apoptotic markers relative to the group that received only the Aβ1‐42 peptide (^####^ *p* < 0.0001). A comparable reduction in apoptosis was observed in cells treated with galantamine prior to Aβ1‐42 exposure (^####^ *p* < 0.0001).

### 3.5 Escin Reduces Aβ1‐42 Mediated Inflammatory Gene Expression in Neuron‐Like Differentiated SH‐SY5Y Cells

**Figure 5:**
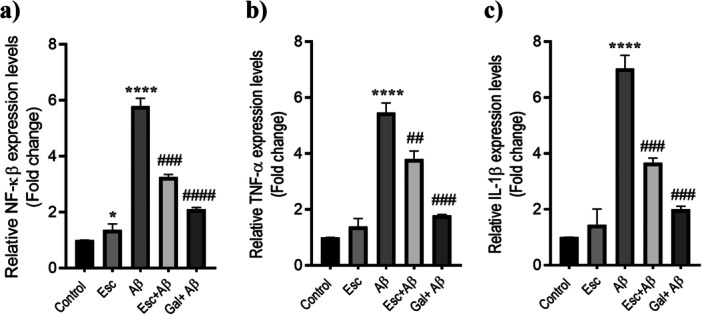
Regulatory effects of Escin on Aβ1‐42‐mediated inflammatory signaling. (a–c) mRNA expression of key inflammatory mediators in neuron‐like SH‐SY5Y cells. The substantial induction of NF‐κB, TNF‐α, and IL‐1β by Aβ1‐42 was partially reversed by Escin pretreatment, showing an efficacy comparable to the reference drug, galantamine. Quantification was performed using RT‐qPCR (normalized to β‐actin). Data are presented as mean ± SD (*n* = 3). *p < 0.05 and ****p < 0.0001 versus Control group; ##*p* < 0.01, ###*p* < 0.001, and ####*p* < 0.0001 versus Aβ1‐42 treated group.

The effects of Aβ1‐42 exposure and Escin pretreatment on inflammatory mediators were assessed by examining NF‐κB, TNF‐α, and IL‐1β mRNA expression levels in neuron‐like differentiated SH‐SY5Y cells. As shown in Figure 5a, Escin alone was associated with a mild but statistically significant increase in NF‐κB expression compared with the differentiated control group (*p < 0.05). Treatment with Aβ1‐42 markedly elevated NF‐κB levels relative to the control (****p < 0.0001). However, Escin pretreatment (Esc+Aβ) and galantamine pretreatment (Gal+Aβ) significantly suppressed Aβ1‐42‐induced NF‐κB overexpression (**###** *p* < 0.001 and **####** *p* < 0.0001, respectively)

Aβ1‐42 administration also resulted in a pronounced increase in TNF‐α mRNA expression when compared with neuron‐like differentiated control cells (****p < 0.0001) (Figure 5b). Pretreatment with Escin or galantamine significantly reduced this elevation (**##** *p* < 0.01 and **###** *p* < 0.001, respectively), compared with the Aβ‐treated group

Similarly, IL‐1β expression was strongly upregulated following Aβ1‐42 exposure compared with the control condition (**** *p* < 0.0001) (Figure 5c). Escin pretreatment substantially decreased IL‐1β levels (**###** *p* < 0.001), while galantamine pretreatment produced a comparable reduction (**####** *p* < 0.0001).

## 4 Discussion

In this study, we evaluated the protective effects of Escin against Aβ1‐42 induced cellular injury in neuron‐like differentiated SH‐SY5Y cells. Escin at a non‐cytotoxic dose of 2 µM (Figure 2b) showed significant cytoprotective activity, likely by reducing oxidative stress, apoptosis, and inflammatory responses. The selection of Escin is supported by its unique multitarget profile; while its lipophilic structure suggests potential brain permeability, its clinical relevance is increasingly linked to its ability to stabilize the neurovascular unit. Recent evidence indicates that Escin protects the blood‐brain barrier (BBB) integrity by upregulating tight junction proteins such as ZO‐1 and Occludin via the AMPK/Cav‐1/MMP‐9 signaling pathway [13, 14]. Furthermore, Escin has been shown to exert neuroprotection indirectly by modulating the gut‐brain axis and preventing the systemic leakage of lipopolysaccharides (LPS), thereby mitigating neuroinflammation even in scenarios where direct CNS penetration might be limited [15, 16]. Galantamine served as a positive control, consistent with previous evidence of its safety and neuroprotective efficacy [22, 23]. While both compounds exhibited comparable efficacy in preserving cell viability, Escin potentially offers a broader multitarget therapeutic window. Unlike Galantamine, which primarily acts as an acetylcholinesterase inhibitor with secondary antioxidant effects, Escin demonstrates a sophisticated disease modifying potential by simultaneously stabilizing the neurovascular unit, modulating the gut‐brain axis, and exerting corticosteroid‐like anti‐inflammatory actions [12, 16]. While 10 µM Aβ1‐42 markedly reduced cell viability, Escin pretreatment preserved cell survival under neurotoxic conditions (Figure 2). These results indicate that our model is suitable for evaluating the neuroprotective potential of Escin.

Within this experimental context, exposure to Aβ1‐42 markedly increased intracellular ROS levels in neuron‐like differentiated SH‐SY5Y cells, supporting the well established concept that Aβ‐induced cytotoxicity is strongly driven by oxidative stress [25, 30]. Escin pretreatment significantly attenuated ROS accumulation, suggesting effective modulation of redox homeostasis. This aligns with previous findings that Escin enhances endogenous antioxidant defenses, including superoxide dismutase, catalase, and glutathione, and mitigates ROS mediated damage in neuronal and oxidative stress models [17, 18, 22, 31, 32]. The positive control, Galantamine, produced a comparable reduction in ROS, in agreement with earlier reports indicating its antioxidative and redox‐stabilizing properties in Aβ related neurotoxicity [33]. However, Escin's antioxidant impact may be more robust in a systemic context, as it not only scavenges radicals but also prevents the influx of exogenous triggers like LPS into the bloodstream a protective feature not typically attributed to Galantamine [16].

Interestingly, Escin alone induced a slight but significant increase in ROS generation. Rather than a purely detrimental effect, this observation likely represents a hormetic pre‐conditioning response, consistent with observations in other cell systems [34], Compelling evidence indicates that Escin can directly interact with Keap1, the primary negative regulator of Nrf2, thereby facilitating the nuclear translocation of Nrf2 and the subsequent transcription of antioxidant‐responsive element (ARE)‐regulated genes, such as HO‐1, NQO‐1, and SRXN‐1 [35, 36]. Recent studies in Aβ‐transgenic models confirm that Escin upregulates the SKN‐1/Nrf2 pathway to counteract oxidative injury and cognitive decline [37]. This adaptive ‘priming’ not only compensates for Nrf2 depletion but also induces autophagy‐related proteins (e.g., Atg5) to maintain cellular proteostasis [12, 38]. Collectively, these findings suggest that Escin enhances cellular resilience through this “molecular priming,” preparing the cells against subsequent Aβ‐mediated toxicity. Although activation of the Nrf2/HO‐1 pathway was not experimentally confirmed in the present study, existing literature strongly supports this mechanism and warrants further investigation

Aβ1‐42 is widely recognized to induce apoptotic cell death in neuronal models, contributing to neurodegeneration in Alzheimer's disease [39, 40, 41]. Consistent with this concept, Aβ1‐42 exposure significantly increased apoptotic cell percentage in neuron‐like differentiated SH‐SY5Y cells, while Escin pretreatment markedly reduced Aβ‐induced apoptosis. This observation accords with previous reports demonstrating the anti‐apoptotic potential of Escin in various cellular systems [18, 42]. Our findings are further supported by recent studies showing that Escin inhibits NLRP3 inflammasome mediated pyroptosis and regulates mitochondrial apoptotic pathways, including the inhibition of Bax upregulation, prevention of cytochrome‐c release, and suppression of caspase activation [14, 43, 44]. This additional layer of protection against programmed cell death provides a sophisticated mechanistic advantage that distinguishes Escin from the primarily cholinergic action of Galantamine [12]. Furthermore, these results, together with earlier studies reporting that Escin attenuates caspase‐dependent apoptosis in oxidative injury models [32], support the notion that Escin can stabilize mitochondrial integrity under neurotoxic conditions. Galantamine also significantly decreased apoptosis with a comparable magnitude, consistent with its previously described antioxidant and cytoprotective effects against Aβ‐induced damage [25]. Interestingly, Escin alone induced a slight but significant increase in apoptosis, suggesting that its effects may be dose‐, context‐, or redox‐state dependent, which should be further clarified in future mechanistic studies. Overall, these data indicate that Escin may confer neuroprotection at least partly through modulation of mitochondria‐associated apoptotic signaling pathways in Aβ‐exposed neuronal cells.

Neuroinflammation, mediated by NF‐κB activation and upregulation of pro‐inflammatory cytokines such as TNF‐α and IL‐1β, represents another key component of Aβ toxicity [45, 46, 47, 48, 49]. Our results show that Escin attenuates Aβ1–42‐induced NF‐κB, TNF‐α, and IL‐1β expression. These effects may, as reported in previous studies, result from either direct modulation of NF‐κB signaling or secondary to its antioxidant and cytoprotective actions [12, 50, 51, 52]. Galantamine exhibited comparable anti‐inflammatory activity, consistent with previous reports [53, 54]. Nevertheless, Escin's suppression of these markers may involve a more direct and potent modulation of the inflammatory cascade. While Galantamine's anti‐inflammatory effects are often secondary to cholinergic enhancement, Escin acts as a direct inhibitor of the NLRP3 inflammasome and the IL‐1β/RhoA/NF‐κB axis, potentially offering more comprehensive protection against chronic neuroinflammation [14, 51]. While we focused on transcriptional regulation, the significant downregulation of NF‐κB mRNA, alongside its downstream targets, strongly indicates functional suppression. Evidence confirms that Escin acts as a competitive modulator of the glucocorticoid receptor, which directly interferes with NF‐κB nuclear translocation and prevents its binding to pro‐inflammatory promoters [12]. Therefore, Escin provides a sophisticated mechanistic advantage by targeting the inflammatory signaling hub at multiple levels.

Our findings of reduced NF‐κB and ROS levels align with literature suggesting that Escin acts as a barrier stabilizer, protecting the structural integrity of the BBB [16]. The observed reduction in Aβ1‐42‐induced neurotoxicity in our model likely reflects Escin's ability to stabilize cellular membranes and modulate downstream inflammatory cascades, regardless of its primary site of action. This multifaceted mechanism targeting both systemic and local pathways provides a sophisticated mechanistic basis for its therapeutic potential in Alzheimer's Disease.

In summary, these findings underscore the neuroprotective potential of Escin, which appears to be mediated by the simultaneous modulation of oxidative homeostasis, the mitochondrial apoptotic cascade, and pro‐inflammatory signaling networks. This study has several limitations. First, the use of a single in vitro neuronal model (SH‐SY5Y cells) and a single Aβ species (Aβ1‐42) may not fully recapitulate the complexity of Alzheimer's disease pathology. Second, the precise molecular mechanisms by which Escin modulates NF‐κB signaling and apoptosis remain to be elucidated. Future studies should explore Escin's effects in primary neuronal cultures, co‐culture systems, and in vivo models of neurodegeneration. Additionally, dose‐response studies and mechanistic investigations on redox‐dependent apoptosis and inflammation will be important to optimize therapeutic potential.

In conclusion, Escin demonstrates promising neuroprotective properties against Aβ‐induced cytotoxicity, attenuating oxidative stress, mitochondrial apoptosis, and inflammatory responses. These results support further investigation of Escin as a potential therapeutic agent in neurodegenerative diseases.

## Author Contributions

**Sakine Akar:** conceptualization, investigation, funding acquisition, writing – original draft, writing – review and editing, methodology, validation, visualization, project administration, resources, formal analysis. **Ozge Alvur:** writing – review and editing, investigation, validation, methodology. **Gulsah Evyapan:** investigation, writing – review and editing, validation, methodology. **Berna Ozdem:** investigation, validation, methodology. **Ilknur Porsuk Doru:** data curation, formal analysis, writing – review and editing, investigation.

## Ethics Statement

The authors have nothing to report.

## Conflicts of Interest

The authors declare no conflicts of interest.

## Data Availability

The data that support the findings of this study are available from the corresponding author upon reasonable request.
